# Early safety and efficiency outcomes of a novel interdisciplinary laparoscopic resection rectopexy combined with sacrocolpopexy for women with obstructive defecation syndrome and pelvic organ prolapse: a single center study

**DOI:** 10.1186/s12893-024-02474-4

**Published:** 2024-06-14

**Authors:** Claudia Rudroff, Joshy Madukkakuzhy, Alberto Vega Hernandez, Jakob Otten, Christoph Ulrici, Leonidas Karapanos, Sebastian Ludwig

**Affiliations:** 1 Department of Visceral Surgery and Functional Surgery of the Lower Gastrointestinal Tract (UGI), Clinic for General and Visceral Surgery, Evangelisches Klinikum Köln Weyertal GmbH, Academic Hospital of the University of Cologne, Weyertal 76, Cologne, 50931 Germany; 2 Department of Obstetrics and Gynecology, Division of Urogynecology and Pelvic Reconstructive Surgery, University Hospital of Cologne and Medical Faculty Cologne, 50931 Cologne, Germany; 3 Department of Urology, Division of Neurourology, University Hospital of Cologne and Medical Faculty Cologne, 50931 Cologne, Germany; 4 Department of General, Visceral and Minimally Invasive Surgery, Park-Klinik Weissensee Berlin, Berlin, Germany; 5 Department of Oral, Maxillofacial and Plastic Surgery, University Hospital Bonn, Bonn, Germany; 6 Department of General and Visceral Surgery, St.Josef Hospital Bonn-Beuel, GFO Kliniken Bonn, Bonn, Germany; 7 Department of Urology, Municipal Hospital of Leverkusen, Leverkusen, Germany

**Keywords:** Obstructed defecation syndrome, Pelvic organ prolapse, Resection rectopexy, Sacrocolpopexy, Synthetic mesh, Biological mesh

## Abstract

**Background:**

Obstructive defecation syndrome (ODS) defines a disturbed defecation process frequently associated with pelvic organ prolapse (POP) in women that substantially compromises quality of life. Conservative management offers limited relief and a surgical intervention may be required. This is characterized by individual approaches.

**Aim of the study:**

This retrospective single center study evaluated the surgical and clinical short-term outcome of a novel interdisciplinary laparoscopic resection rectopexy (L-RRP) with mesh- sacrocolpopexy (L-SCP) for women suffering from ODS and POP.

**Methods:**

The study participants underwent surgery in an interdisciplinary laparoscopic approach. Safety was the primary endpoint, assessed via postoperative morbidity classified by Clavien-Dindo scale. Secondary outcomes included evaluation of bowel function, fecal and urinary incontinence and pelvic organ prolapse status at 12 months follow-up. Additionally, a biological mesh (BM) was offered to women, who asked for an alternative to synthetic mesh material (SM).

**Results:**

Of the 44 consecutive patients requiring surgery for ODS and POP, 36 patients underwent the interdisciplinary surgical approach; 28 patients with SM and 8 patients with BM. In total 5 complications occurred, four of them were classified as minor. One minor complication was observed in the BM group. One anastomotic leakage occurred in the SM group. The two ODS scores, the bowel dysfunction score, and the incontinence score improved significantly (*p* = 0.006, *p* = 0.003, *p* < 0.001, and *p* = 0.0035, respectively). Pelvic floor anatomy was fully restored (POP-Q 0) for 29 (80%) patients after surgery. 17 patients (47%) suffered from urinary incontinence before surgery, which was restored in 13 patients (76.5%).

**Conclusions:**

The interdisciplinary approach with L-RRP and L-SCP and the use of a BM in a small subgroup were technically feasible, safe, and effective in this single center setting. The study’s retrospective design, the small sample size and the lack of comparators limit the generalizability of the findings requiring future randomized trials.

**Trial registration:**

Retrospectively registered at clinicaltrials.gov, trial number NCT05910021, date of registration 06/10/2023.

## Background

Obstructive defecation syndrome (ODS) summarizes a disturbed defecation process and is often caused by a rectocele, an internal rectal prolapse—a sort of telescoping of the rectal wall within itself (intussusception), or a full rectal prolapse [1–5]. The patients must exert pressure to evacuate the rectum and sometimes require manual assistance [6]. The unsuccessful attempts to defecate are associated with a feeling of incomplete rectal voiding. ODS affects a significant proportion of the population (10–25%), with a higher prevalence in women, and is often associated with pelvic organ prolapse (POP) [7]. Almost 60% of the female population develop POP during their lifetime but just 1/3 of them suffer bowel dysfunction [8, 9]. Individual aspects, such as pregnancy, childbirth, connective tissue disorders, and pelvine surgical interventions further contribute to the condition [7, 10, 11]. The women experience frustration due to their disturbed defecation causing a profound negative impact on their quality of life. Conservative treatment options are limited and often do not achieve the desired long-term effect [12, 13]. Almost 20% of the women require surgery during their lifetime [14, 15].

The surgical treatment aims at the anatomic reconstruction of the bowel and pelvic floor and has been characterized by individual approaches [16]. The complexity of the underlying condition is often not addressed by all disciplines and the anticipated results often fail [13, 17].

The European consensus statement on the treatment of ODS published in 2021 [18] evaluated a wide variety of diagnostic and treatment aspects. A standardized treatment option for evidence-based advice to women suffering from ODS in combination with POP is still missing [19–22]. And only scarce data on interdisciplinary approaches exist to date [23, 24].

Another important subject in reconstructive pelvic floor surgery is the controversial discussion on the use of synthetic mesh (SM) material due to their potential risk of severe complications. SMs inherit the risk of acute and chronic infection, erosion, and migration which may lead to serious consequences as a lifelong diverting stoma years after surgery [22, 25–31]. Therefore, the use in transvaginal surgery was restricted by the Food and Drug Administration (FDA) in the United States [25, 27, 32, 33]. At the same time the surgical treatment of POP with SCP the SM material is still the standard of care according to the German gynecology guideline on pelvic surgery. The use of a biological mesh (BM) is explicitly not recommended, although the underlying scientific evidence is scarce [21, 34–37]. On the other hand, in colorectal surgery the use of a BM as an alternative to a SM has already proven its safety and efficacy in laparoscopic ventral mesh rectopexy and has earned FDA approval [19, 20, 38, 39]. The missing long-term data on the use of meshes, especially from synthetic material, may overstate the benefits and understate the risks with its application. Further evidence is urgently needed.

This study evaluated the feasibility, safety and short-term outcome of an interdisciplinary surgical approach of laparoscopic resection rectopexy (L-RRP) combined with a mesh sacrocolpopexy (L-SCP as a synonym for apical mesh fixation of the middle compartment to the sacral promontory). Furthermore, an absorbable BM for L-SCP was offered those women who asked for an alternative to the synthetic mesh (SM) and to those who wished to preserve the uterus for a later planned pregnancy.

## Methods

### Aim, design and setting of the study

The aim of this study was to assess the feasibility, the safety and the short-term outcome of a novel surgical approach combining laparoscopic RRP with SCP in an interdisciplinary setting.

Between February 2020 and August 2021 a cohort of 44 consecutive women suffering from ODS and POP presented to our tertiary pelvic floor center (POC) to evaluate the need of surgical therapy. After thorough diagnostic work up (see below) all cases were routinely presented and discussed at the POC board. The recommended treatment as well as alternative treatment options, including enhanced conservative therapy and various other surgical approaches, were discussed thoroughly. A group of 36 patients were indicated for L-RRP combined with L-SCP and included in this study. A BM as an alternative to the standard synthetic mesh was offered those patients, who expressed their concern about the standard SM material and who explicitly asked for an alternative as described above.

For all 36 participants written informed consent for the interdisciplinary approach, the mesh material used, the data collection, and the publication of the results was obtained before surgery. Thereafter, the procedure was scheduled.

The data was collected prospectively; the analysis was carried out retrospectively.

**Fig. 1:**
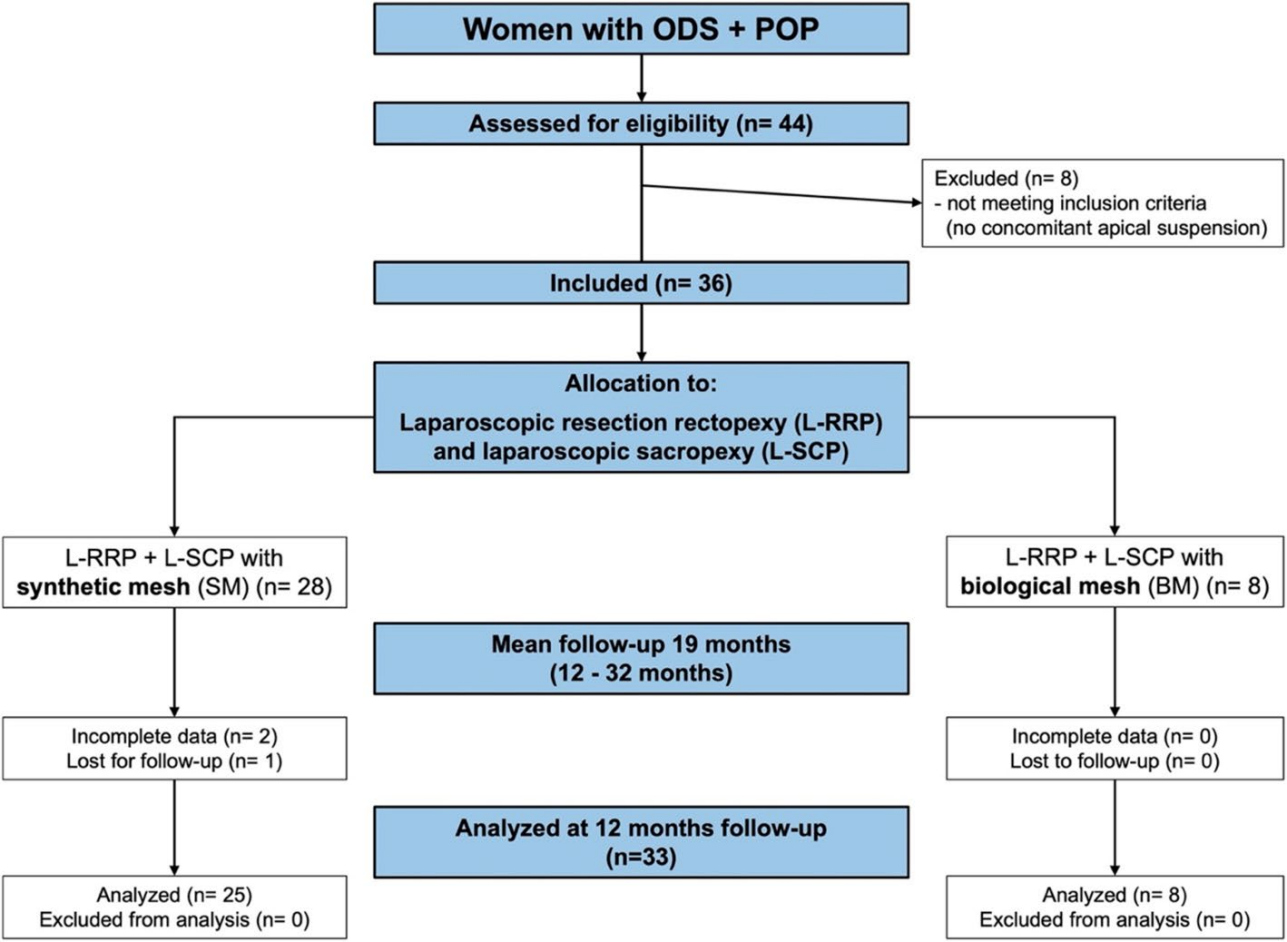
Flowchart of the study. 44 women suffering from obstructed defecation syndrome (ODS) and pelvic organ prolapse (POP) presented to the tertiary pelvic floor center after failure of conservative treatment. All 36 patients, who received laparoscopic resection rectopexy (L-RRP) and sacropexy (L-SCP) were enrolled in the study. 28 patients received a synthetic mesh (SM) for the L-SCP; in 8 patients a biological mesh (BM) was used

The patients included had to be > 18 years of age and eligible for laparoscopic surgery. Pregnancy or a known allergy against mesh material were exclusion criteria (Fig. 1).

#### Characteristics, clinical and anatomical scores

Patients’ characteristics as age, body mass index (BMI), and comorbidity as represented by the ASA score (American Society of Anesthesiologists, ASA) [40] were documented.

Upon presentation and at 12 months after surgery all patients underwent urogynecological and surgical examinations at the outpatient clinic. Clinical results were augmented with validated questionnaires on various bowel function symptoms.

Obstructed defecation symptoms were measured with two validated ODS questionnaires, the Altomare score (maximum 30 points) and the modified Longo ODS Score (maximum 24 points) [41, 42].

Bowel dysfunction, such as diarrhea, meteorism, spasm, bleeding, and abdominal pain during bowel movement and defecation were assessed with the rectal toxicity score (maximum 32 points), a validated score for colorectal symptoms for patients after radiation therapy of the pelvis for prostate or gynecologic cancer [43–46].

Accompanying stool incontinence symptoms were documented with the Wexner incontinence score (maximum 20 points) [47]. For all questionnaires higher scores identify more severe symptoms, however, no validated cut-off levels exist.

Evaluation of the type and degree of the pelvic organ prolapse (POP) applied the POP quantification system (POP-Q) [48].

### Diagnostic work up

**Fig. 2:**
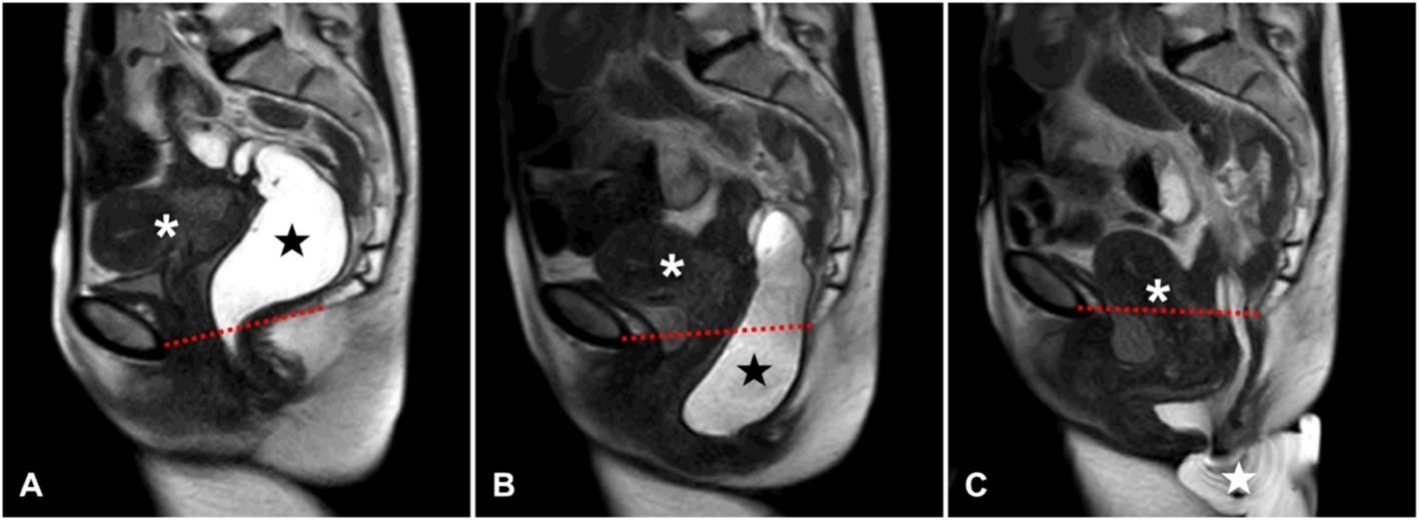
Magnetic resonance image defecography (MRI-D) in sagittal view of a female pelvis with obstructed defecation syndrome (ODS) and pelvic organ prolapse (POP) before surgery. The red dotted arrow marks the pubo-coccygeal line. The white asterisk marks the uterus, the black star marks the filled rectum (ultrasound gel), and the white star marks the excreted ultrasound gel. Figure 2A shows the position at rest, 2B shows the defecation process and descending of the rectum and uterus, and 2C shows the excreted ultrasound gel

All patients included in this study were examined by both a trained colorectal surgeon and a uro-gynecologist, as stated above. The magnetic resonance image defecography (MRI-D) as shown in a representative case in Fig. 2 (A, B, and C) was obligatory for all cases before surgery.

The results from the clinical examination and the magnetic resonance imaging served to define the anatomical defect causing the ODS classifying the findings into a rectocele, an enterocele, a rectal intussusception, and a full rectal prolapse.

All cases presenting to our POC were routinely presented and discussed at the POC board. There was a minimum of 4 weeks between the first presentation, the completion of diagnostics, and the planned treatment in cases of surgery. No patient was rushed to their individual decision and care was taken to ensure a fully consented decision from the patient’s point of view.

### Endpoints of the study

The primary study outcome parameter was the safety performing the combined laparoscopic procedure and was assessed through postoperative morbidity and mortality measured by the Clavien-Dindo classification (CDC) [49].

The secondary outcome parameters were clinical and anatomical outcomes as measured by the aforementioned scores. All scores were routinely collected before the surgery and at the follow-up examination at 12 months after surgery.

### Surgical procedure and mesh material

**Fig. 3:**
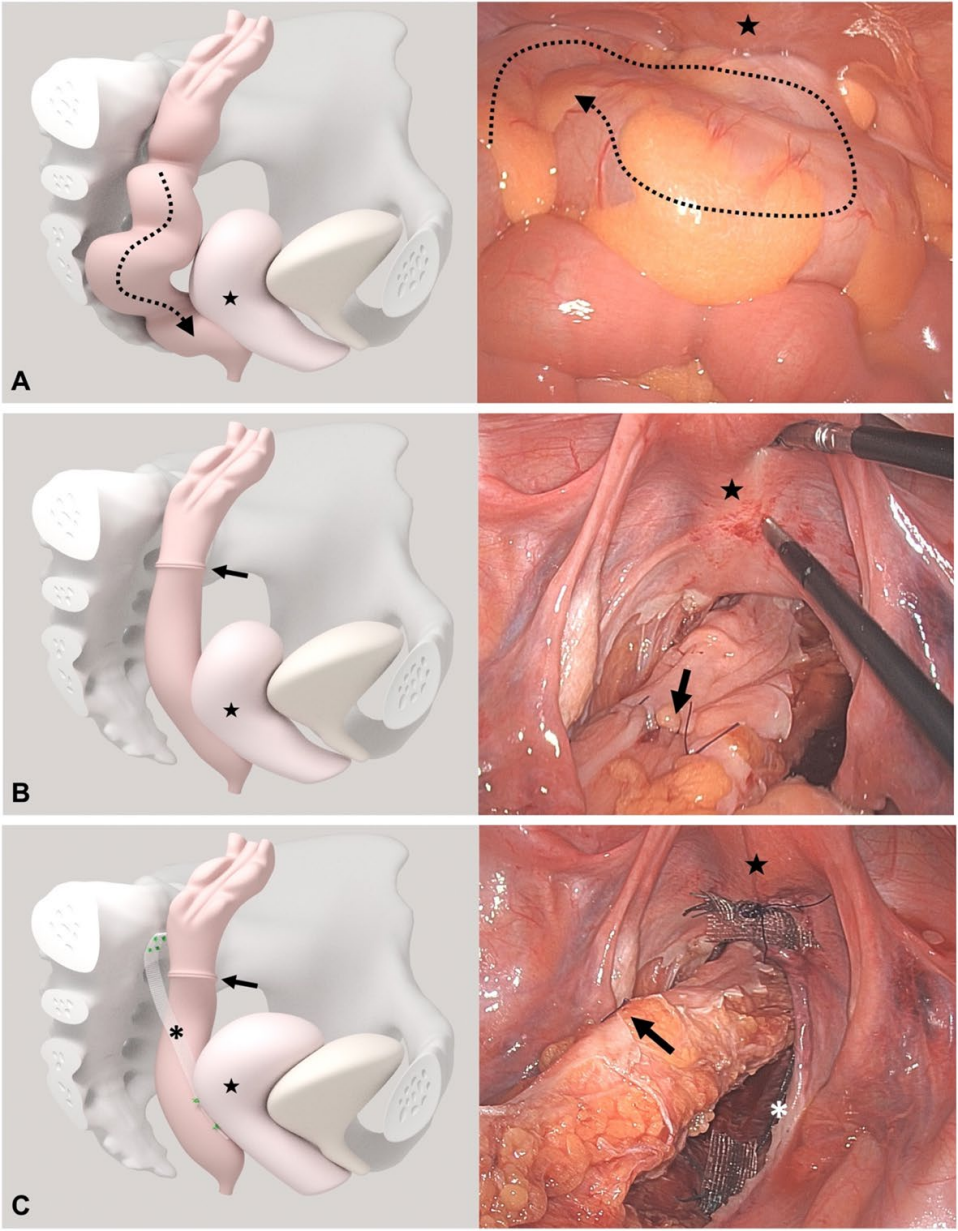
Sagittal view of the female pelvis with obstructed defecation syndrome (ODS) and pelvic organ prolapse (POP) before and after the combined laparoscopic resection rectopexy (L-RRP) and sacrocolpopexy (L-SCP) as well as intraoperative laparoscopic pictures. **A.** The black dotted arrow marks the elongated and descending sigma, and the black star marks the descending uterus before the combined surgical procedure. The right picture shows the elongated sigma completely filling the small pelvis. **B.** The black arrow marks the sigma anastomosis after resection, but before rectopexy. The black star marks the descending uterus. **C.** The black arrow marks the sigma after L-RRP. The black asterisk marks the synthetic mesh for L-SCP, in the depict case a hysteropexy. On the laparoscopic picture a white asterisk marks the synthetic mesh. The mesh is placed unilaterally on the right side of the small pelvis, fixed at the posterior cervix and the sacrum at the level of promontory / S1. The black star marks the elevated uterus after apical fixation

For the laparoscopic procedure in general anesthesia, patients were maintained in dorsal lithotomy position and head-down position of 18°. CO_2_ pneumoperitoneum was established according to institutional standards, and trocars were placed. The procedure started with the preparation of the rectum. The opening of the retrorectal Waldeyer space and the rectovaginal space ensured the complete mobilization of the rectocele and the rectal intussusception down to the pelvic floor. After the aboral resection margin was marked a tubular anterior rectal resection and the removal of the elongated sigmoid with its functional kinking were performed (Fig. 3A and B). The bowel continuity was reconstructed with an end-to-end descendo-rectostomy using a circular stapling device (29 mm Endoscopic Curved Intraluminal Stapler, Johnson & Johnson Medical GmbH, Robert-Koch-Strasse 1, 22,851 Norderstedt, Germany). A suture rectopexy fixed the rectum to the sacral vertebrae at the left side of the promontory with nonabsorbable sutures.

The apical fixation of the middle pelvic organ compartment was performed unilaterally in analogy to the previously published cervicosacropexy and vaginosacropexy technique (Fig. 3C) [50, 51]. The comparable procedure fixing the cervix with a mesh was used in cases the uterus was preserved.

The synthetic mesh material (SM) consisted of polyvinylidene-fluoride (DynaMesh VASA, FEG Textiltechnik GmbH Aachen, Germany). In selected cases as described above a biological mesh (BM) (Biodesign® Rectopexy Graft, Cook Biotech Incorporated, USA) substituted the SM. A pelvine peritoneoplasty with resorbable sutures covered the mesh material and obliterated the small pelvis and the Douglas pouch.

### Data management and statistical analysis

The required clinical data were collected preoperatively, during the hospital stay, and during the follow-up examinations. All scores were documented on paper and transferred to a data bank. Data were analyzed using the SPSS statistical package, version 29.0.0.1. (IBM Corp., Armonk, NY, USA). Quantitative variables are described as means (± standard deviation) and were compared using the Kruskal–Wallis H test and Mann–Whitney U test. Qualitative variables are summarized using count, percentage, median, and interquartile range and were compared using the Fisher’s exact test. A two-sided p value of  < 0.05 was considered statistically significant. Because no adjustments for multiple testing were performed, the analyses were exploratory.

## Results

Of the 44 women with ODS and POP treated with surgery, eight patients were excluded from further analysis due to transvaginally reconstruction of the pelvic floor. 36 patients underwent operation with the novel interdisciplinary approach combining L-RRP with L-SCP as described above. The median follow-up period was 25 months (12–36 months) with 35 patients to be analyzed at 12 months. For L-SCP a SM was used in 28 patients. A BM was chosen in 8 patients due to patients’ preference or a planned pregnancy. One patient in the SM group was lost to follow-up. In two patients of the SM group the 12 months data were incomplete for the bowel function scores and were marked as missing values for statistical analysis of the follow-up.

### Patients’ characteristics

Patients’ median age was 57.5 years (range: 26–83 years), and the mean body mass index (BMI, kg/m^2^) was 23 (18–35) with no statistically significant differences between the two subgroups, SM and BM. The ASA score ranged from ASA I (*n* = 2) to ASA III (*n* = 12), with most of the patients in the ASA II subgroup (*n* = 22). Patients in the BM group classified in ASA I (*n* = 1) and ASA II (*n* = 7). In the SM group 12 patients were ASA III. The difference was statistically significant (*p* = 0.029).

The anatomical defect causing the obstructed defecation symptoms of the participants were a rectocele in 34 cases (94.4%), an intussusception in 23 patients (63.9%), an enterocele with 16 patients (44.4%), and a full rectal prolapse in 2 patients (5.5%). Multiple diagnoses were possible, with 6 patients (16.7%) suffering from a rectocele, only, 19 patients (52.8%) having two defects – mostly rectocele and a rectal intussusception, and 11 patients (30.6%) with three defects. The two patients with a full rectal prolapse suffered from an enterocele, as well. In the BM group all patients suffered from a rectocele, 3 patients (37.5%) had an accompanying rectal intussusception, and one patient (12.5%) an additional enterocele. In the SM group 26 patients (92.9%) had a rectocele, 20 patients (71.4%) a rectal intussusception, and 15 patients (53.6%) had an enterocele. The two patients (7.1%) with full rectal prolapse were in the SM group, as well. The underlying anatomical defect differed between the two subgroups significantly (*p* = 0.031).

Table 1 summarizes the baseline clinical characteristics of the operated patients.

**Table 1:** Patient’s characteristics of the 36 patients with obstructive defecation syndrome (ODS) and pelvic organ prolapse (POP) operated with combined laparoscopic resection rectopexy (L-RRP) and sacrocolpopexy (L-SCP). P-Values showed a significant difference between the two subgroups for the ASA classification. The anatomical defect causing the ODS are listed in four categories; rectocele, rectal intussusception, enterocele, and full rectal prolapse. Multiple findings were possible and are all counted

	**all patients** **(*n* = 36)**	**biomesh** **(*n* = 8)**	**synthetic mesh** **(*n* = 28)**	***p*-value**
Age, y, median (IQR)	57.5 (26 – 83)	47 (26 – 77)	59 (37 – 83)	0.189
ASA, n (%)				0.029
I	2 (5.6%)	1 (12.5%)	1 (3.6%)	
II	22 (61.1%)	7 (87.5%)	15 (53.6%)	
III	12 (33.3%)	0	12 (42.9%)	
BMI (kg/m^2^), median (IQR)	23 (18 – 35)	23.5 (20 –31)	23 (18 – 35)	0.646
Anatomical defect (77 findings in 36 patients)				
Rectocele				0.031
Intussusception				
Enterocele	34 (94.4%)	8 (100%)	26 (92.9%)	
Rectal prolapse	23 (63.9%)	1 (12.5%)	20 (71.4%)	
	16 (44.4%)	3 (37.5%)	15 (53.6%)	
	2 (5.5%)	none	2 (7.1%)	

Distributions are presented as median and interquartile range (IQR) for continuous, and total number and percentages (%) for binary data

### Surgical outcome

All patients were operated laparoscopically with no conversion to open surgery. The average operation time was 263 min (interquartile range (IQR) 165–418 min) and postoperative average hospital stay was 8.2 days (min/max 4–15 days), with no statistical difference between the subgroups (Table 2).

**Table 2:** Surgical outcome of the patients including operating time and duration of hospital stay. P-Values show no difference between the two subgroups

	**all patients** (***n*** = 36)	**biomesh** (***n*** = 8)	**synthetic mesh** (***n*** = 28)	**p-value**
Operation time in min, median/IQR	263 (165 – 418)	245 (165 – 331)	264 (177 – 418)	0.253
Duration of hospital stay in days, mean (min/max)	8.2 (4–15)	8.4 (5–11)	8.2 (4–15)	0.671

Distributions are presented as median and interquartile range (IQR) for continuous, and total number and percentages (%) for binary data

### Morbidity and mortality

In total, postoperative complications occurred in 5 cases. Most of the complications were categorized as mild (CDC 1–3a). Superficial wound infection (CDC 1) in one patient of the BM group required no further treatment. One urinary tract infection, a postoperative episode of diarrhea in one patient, and one lung embolism 5 days after surgery with mild symptoms were all treated with medication (CDC 2). One patient in the SM group developed an anastomotic leakage and required surgical revision with a temporary loop ileostomy (CDC 3b). Further recovery was uneventful, and the patient was discharged on day 10. The complication risk was similar after biological and synthetic mesh implantation (1/8 vs. 4/28; relative risk 0.88; 95% confidence interval 0.11 to 6.8). For further details see Table 3.

**Table 3:** Patient’s morbidity and mortality according to the Clavien Dindo Classification (CDC) was listed and further stratified into minor complications (CDC 1-3a) and major complications (CDC 3b-5). Furthermore, the individual complications were counted and classified clinically

	**all patients** (***n*** = 36)	**biomesh** (***n*** = 8)	**synthetic mesh** (***n*** = 28)	***p***-value
Overall morbidity, n (%)	5 (13.9%)	1 (12.5%)	4 (14.2%)	0.170
Minor (CDC 1 – 3a), n (%)	4 (11.1%)	1 (12.5%)	3 (10.7%)	
Major (CDC 3b – 5), n (%)	1 (2.8%)	0	1 (3.6%)	
Mortality, n (%)	0	0	0	
Anastomotic Leakage	1 (2.8%)	0	1 (3.6%)	
Lung embolism	1 (2.8%)	0	1 (3.6%)	
Wound infection	1 (2.8%)	1 (12.5%)	0	
Gastrointestinal	1 (2.8%)	0	1 (3.6%)	
Urologic	1 (2.8%)	0	1 (3.6%)	

Distributions are presented as median and interquartile range (IQR) for continuous, and total number and percentages (%) for binary data

### Clinical and functional outcome after surgery

#### Obstructed defecation syndrome (ODS) and bowel function

**Fig. 4:**
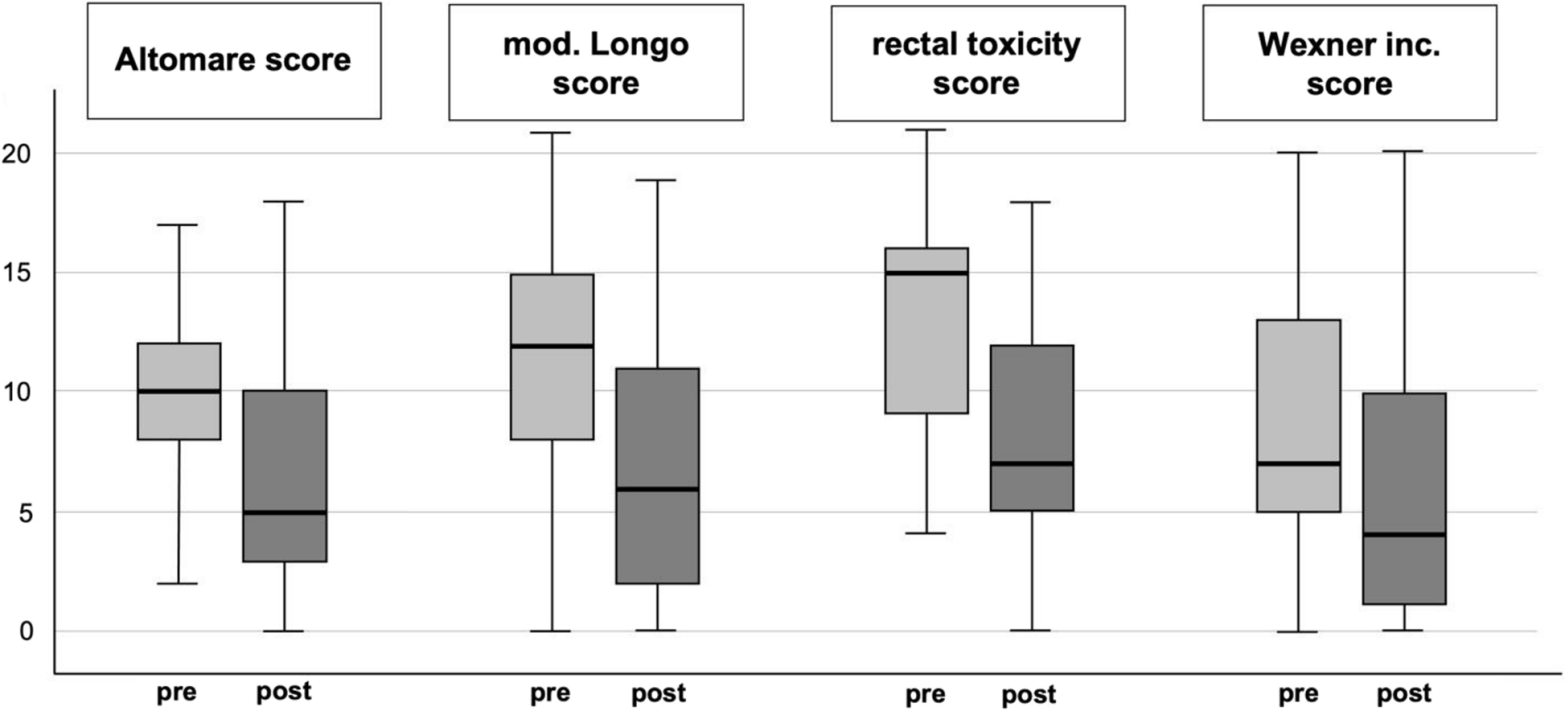
The summarized results of the clinical bowel symptoms of the 36 patients with respect to the obstructed defecation syndrome (ODS), the functional bowel symptoms, and fecal incontinence are visualized below. The y-axis represents the numeric value of the score in 5-point steps. The boxplots are showing the median score values of the questionnaires for the Altomare score, the modified Longo ODS score, rectal toxicity score, and Wexner incontinence score depicted as boxplots pre- and postoperative (at 12 months

The ODS symptoms were classified clinically by using scores before and after surgery. The Altomare score (maximum 30 points) averaged 10 points (2–24) preoperatively and dropped to 5 points (0–18) at 12 months follow up (*p*= 0.006) (Fig. 4). In the SM group the score dropped from 10 (0–19) preoperatively to 6 (0–18) after surgery; the difference was statistically significant (*p* = 0.023). The categorial analysis showed an improvement for the BM group, as well. Before surgery the BM group scored 10.5 (3–24). The score dropped to 4.5 (2–17), which did not reach statistical significance due to the small sample size (*p* = 0.074).

The mean results in the modified Longo score (maximum 24 points) reached 12 points (2–20) preoperatively and 6 points (0–19) after 12 months (*p* = 0.003) (Fig. 4). For the SM group the results between the preoperative value 12.5 (0–21) and the 12 months follow up of 6 (0–18) differed significantly (*p* = 0.015). The changes in the BM subgroup between 11.5 (6–10) before and 7.5 (2–17) 12 months after surgery were not statistically significant (*p* = 0.09).

Bowel dysfunction symptoms were measured using the rectal toxicity score (maximum 32 points). In the total cohort, 15 points (4–21) were measured preoperatively and 7 points (0–17) at 12 months after surgery. The improvement after surgery was statistically significant (*p* < 0.01) (Fig. 4). Both subgroups improved significantly after surgery, as well. The median value in the SM group changed from 15 points (4–21) before surgery to 8 points (0–18) at follow up (*p* = 0.004). The results in the BM group showed lower values for both time points, 10 points (5–18) and 6 points (3–17), respectively (*p* = 0.04).

**Fig. 5:**
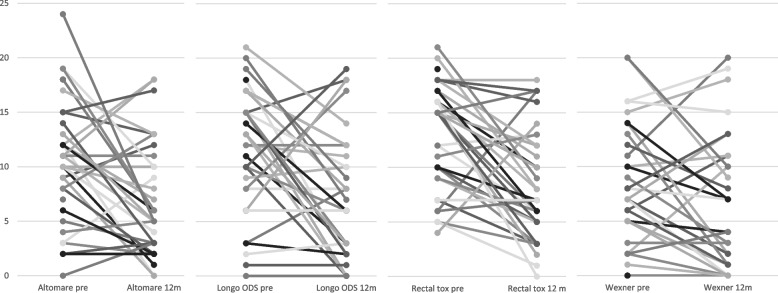
The distribution of each individual score result for the Altomare score, the modified Longo ODS score, rectal toxicity score, and Wexner incontinence score before surgery and after 12 months follow up are shown in a linear diagram. The y-axis represents the value of the score in 5-point-steps. Each line represents one patient to give an impression of the individual outcome after the interdisciplinary surgical approach addressed in the study

The Wexner incontinence score as a parameter for the control of flatus and stool improved after surgery. Preoperatively the patients scored 7 points (0–20), which dropped to 4 points (0–20) at 12 months (*p* = 0.035) (Fig. 4). The results differed significantly between the subgroups, as well. In the SM group, the median was 8 points (0–20) before and 4 points (0–20) after surgery. The difference was statistically significant (*p* = 0.01). The scores in the BM group, 2 points (0–16) before and 3 points (0–15) after surgery, did not reach statistical significance (*p* = 0.5). All results are summarized in Table 4. Furthermore, the results were depicted in Fig. 4 as box plots with median, interquartile range and min/max values. To visualize the individual changes for each patient Fig. 5 shows a line diagram with each value of each score before surgery and at 12 months follow up.

**Table 4:** The results of the clinical bowel scores for ODS symptoms, functional bowel symptoms and fecal incontinence for the total study cohort as well as for the two subgroups with the synthetic mesh material and the biological mesh are listed with median values (minimum and maximum) and the p-value after statistical analysis

	**All patients** (***n*** = 36)			**biomesh** (***n*** = 8)			**synthetic mesh** (***n*** = 28)		
Scores	Before surgery	12 months	*p*- value	Before surgery	12 months	*p*- value	Before surgery	12 months	*p*- value
Altomare median (min/max)	10 (0–24)	5 (0–18)	0.006	10.5 (3–24)	4.5 (2–17)	0.074	10 (0–19)	6 (0–18)	0.023
Longo ODS median (min/max)	12 (2–20)	6 (0–19)	0.003	11.5 (6–19)	7.5 (0–19)	0.09	12.5 (0–21)	6 (0–18)	0.015
Rectal toxicity median (min/max)	15 (4–21)	7 (0–17)	< 0.001	10 (5–18)	6 (3–17)	0.04	15 (4–21)	8 (0–18)	0.004
Wexner median (min/max)	7 (0–20)	4 (0–20)	0.035	2 (0–16)	3 (0–15)	0.5	8 (0–20)	4 (0–20)	0.01

Distributions are presented as median and minimum and maximum values

#### Pelvic organ prolapse (POP)

**Fig. 6:**
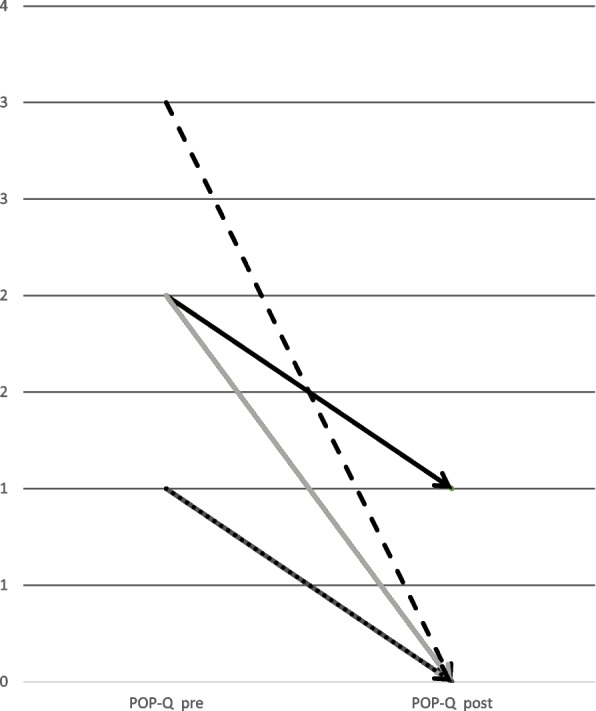
The value of the POP-Q score result before surgery and after 12 months follow up are shown in a linear diagram to give an impression of the outcome after the interdisciplinary surgical approach addressed in the study. The y-axis represents the value of the POP-Q score reaching from 0 to 4. The three patients with no change in the POP-Q score (horizontal lines) are not shown. The dotted line from POP-Q 3 drops to POP-Q 0 after 12 months represents one patient. Three patients in the SM group were improved, but not totally restored with respect to their POP (POP-Q 2 before surgery to POP-Q 1 after 12 months). No surgical re-intervention was wished for. The line POP-Q 1 to POP-Q 0 represents 12 Patients and the line POP-Q 2 to POP-Q 0 16 patients, who were all totally restored with respect to their POP at 12 months follow up.

Postoperative anatomical results were compared with preoperative evaluation by using the POP-Q score. Before surgery, 14 patients (38.9%) had POP-Q 1, 21 patients (58.3%) had POP-Q 2, and one patient had POP-Q 3. Postoperatively, 29 (80%) patients had POP-Q 0. Four patients with POP-Q 2 before surgery improved to POP-Q 1. Two patients with POP-Q 1 did not improve after surgery. One patient with POP-Q 2 had an early relapse after 6 months due to insufficient fixation of the BM. She underwent laparoscopic apical refixation with an SM. The difference between these results was statistically significant (*p* < 0.001). To depict the results more clearly, they are listed in Table 5 below. Furthermore, Fig. 6 serves to illustrate the individual result for each patient in a diagram.

**Table 5:** The results of the POP-Q for the total study cohort as well as for the two subgroups with the synthetic mesh material and the biological mesh are listed with mean values (minimum and maximum) and the *p*-value after statistical analysis. Furthermore, the number of patients in each POP-Q stage preoperatively and after 12 months follow up are listed below

	**All patients** (***n*** = 36)			**biomesh** (***n*** = 8)			**synthetic mesh** (***n*** = 28)		
	Before surgery	12 months	*p*- value	Before surgery	12 months	*p*- value	Before surgery	12 months	*p*- value
POP-Q mean (min/max)	2 (1–3)	0 (0–2)	< 0.001	2 (1–2)	0 (0–2)	0.001	10 (1–3)	6 (0–1)	< 0.001
I, n(%)	14 (38.9%)	6 (16.7%)		5 (62.5%)	1 (12.5%)		9 (32.1%)	5 (17.9%)	
II, n(%)	21 (58.3%)	1 (2.8%)		3 (37.5%)	1 (12.5%)		18 (64.3%)	0	
III, n(%)	1 (2.8%)	0		0	0		1 (3.6%)	0	

Distributions are presented as mean and minimum and maximum values and number of patients in each subgroup

#### Urinary incontinence

**Fig. 7:**
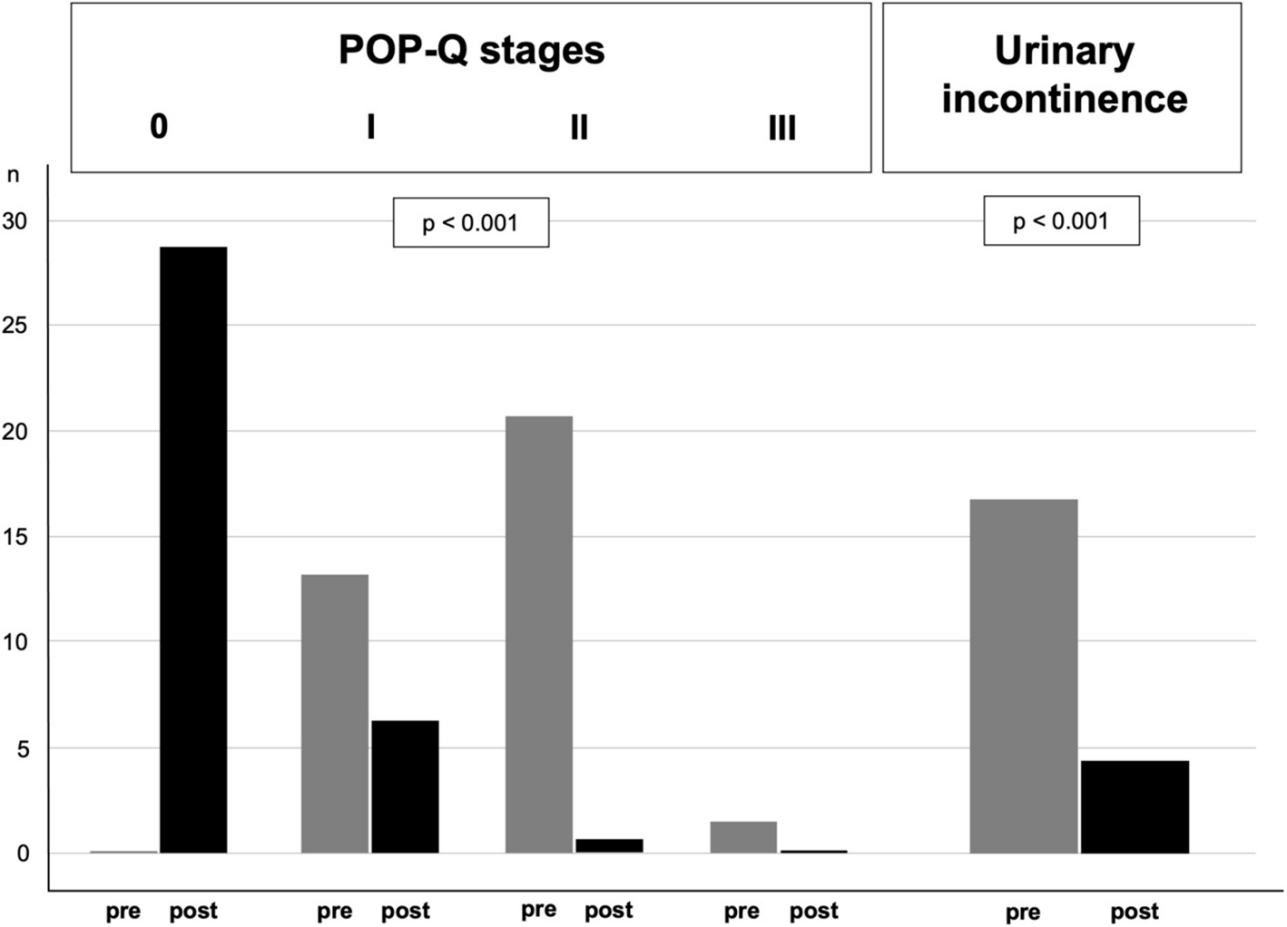
The clinical outcome for pelvic organ prolapse quantification system (POP-Q) stages (0 – IV) and urinary incontinence of the 36 patients are illustrated in a block diagram. The results are depicted pre- and postoperatively at 12 months follow-up after surgery

Of all 36 patients who underwent L-RRP and L-SCP, 17 (47%) patients suffered from urinary incontinence before surgery (mixed urinary incontinence), one patient in the BM subgroup and 16 patients in the SM group. Postoperatively continence was restored in 13 patients (76.5%). No de-novo stress or urgency urinary incontinence symptoms appeared. The results were statistically significant (*p* < 0.001, Fig. 7). In the subgroup analysis the urinary incontinence was restored for 12 patients in the SM group, the improvement was statistically significant (*p* < 0.001). In the BM group urinary incontinence was restored for the one patient suffering from it. To visualize the changes clearly, Fig. 7 plots the results for urinary incontinence together with the POP-Q scores in a block diagram below.

## Discussion

To date, the treatment of patients suffering from ODS combined with POP has been characterized by individual approaches. For ODS alone surgical experts from all over Europe came to a mutual agreement and defined a European consensus that was published in 2021 [18]. However, sufficient data on standardized treatment options to give evidence-based advice to women with ODS and POP—particularly in a combined interdisciplinary approach—is missing [19–22, 24, 52].

This study evaluates an interdisciplinary laparoscopic surgical approach, combining a L-RRP (with anastomosis and without protective stoma) and a L-SCP in one surgical procedure as a promising treatment option for women with ODS and POP.

Both, anatomical and functional outcomes were evaluated after surgery in this novel interdisciplinary setting at our institution. The procedure proved to be technically feasible, safe, and easy to establish. The morbidity was low—with only one surgical re-intervention in the SM group due to an anastomotic leakage requiring a temporary stoma. The synthetic mesh was not adjacent to the leakage and remained in situ. No further complications occurred within 24 months after surgery.

Satisfactory functional outcomes for ODS were achieved as shown in a significant improvement of the assessed scores. Additional bowel discomforting symptoms, as diarrhea, meteorism as well as fecal incontinence were also improved as a positive side effect. Furthermore, good anatomical outcomes were obtained after this surgical procedure with a significantly improved POP in 82% and urinary incontinence in 77% of the patients.

Of the 40 registered clinical trials evaluating the treatment of the medical condition, either ODS and/or POP, not a single trial includes an interdisciplinary surgical approach. The promising results of our single center study show the safety, feasibility, and efficacy in functional results for the women at risk and should be considered as a reasonable treatment option.

The use of a synthetic mesh in pelvic floor surgery inherits the risk of acute and chronic infection, erosion, and migration which may lead to serious consequences as a lifelong diverting stoma years after surgery [22, 25–31]. Consequently, its use in transvaginal surgery was restricted by the Food and Drug Administration (FDA) of the United States [25, 27, 32, 33]. The use of a BM as an alternative has already proven its safety and efficacy in laparoscopic ventral mesh rectopexy and has FDA approval [19, 20, 38, 39]. According to the German guidelines for the surgical treatment of POP with SCP a BM is currently not recommended, although the underlying scientific evidence is scarce [21, 34–37].

In this study a BM was offered for apical fixation in selected cases, as a planned pregnancy and if required by the patient. Postoperative morbidity was similar and the functional results did not differ from those with the synthetic material, although numbers were too small for definitive conclusions. Considering the lifelong risk of a SM and the preliminary results showing safety and efficacy of a BM in this study, it should be considered as an alternative option, particularly in surgery of younger women and in combination with a simultaneous colorectal resection (L-RRP). Because of the preliminary results in this study a prospective randomized pilot study comparing the use of a BM with the SM was initiated [53]. This study should acquire more conclusive and robust results on this important matter.

Nonetheless, the study has some limitations, that affect the strength and applicability of its conclusions. The small number of patients and the retrospective design limit the generalizability of the drawn conclusions, which is why we initiated a prospective randomized trial on this topic [53].

Additionally, the lack of a control group or a comparison with other surgical techniques does not allow for a direct assessment of the novel approach’s superiority nor equivalence in this setting. However, due to the unique interdisciplinary approach that encompasses the entire treatment process from diagnosis to therapy, it is difficult to define a suitable control group. Conservative treatment cannot be compared to surgery, which is even more the case, since all included patients underwent conservative treatment prior to surgery – which no effect anymore.

Furthermore, a technique, that addresses only one aspect of the medical condition – ODS or POP – cannot serve as a suitable comparator to a surgical approach combining two interventions in an interdisciplinary approach.

Another drawback was the short follow-up period of 12 months. The early outcome results are not suitable to draw final conclusions and establish the method as standard of care. Here, the analysis of long-term results from a larger cohort from the study center will bring further evidence about the efficacy and durability of the surgical procedure, shortly.

The choice of mesh material is another critical aspect of the study. The small subgroup of patients in the BM group, the selection bias concerning the decision to choose a BM, which was strictly patient driven, and the short-term follow up leave open questions with respect to the safety profiles and durability of this mesh material. On the other hand, the safety profile of the synthetic mesh material, which is under controversial discussion due to potential severe complications, cannot be answered from this small patient group and the short-term results. Without long-term data, there is a risk that the benefits may be overstated, and the risks understated**.** And despite the early results for the BM, that offer an additional uterus-preserving treatment option, particularly for younger women of child-bearing age and in times of FDA warnings, they are not suitable to draw definitive conclusions yet. Consequently, we initiated a prospective randomized trial on the use of the mesh material, which is already recruiting patients and should timely shed light on this matter [53].

## Conclusions

This study is the first known trial combining an interdisciplinary laparoscopic resection rectopexy with sacrocolpopexy as a simultaneous treatment option for women with ODS and POP. In selected cases women were offered a BM as an alternative to the standard SM for L-SCP with comparable results, so far. This surgical approach in this single center study was technically feasible and showed promising safety and early follow-up results. Long-term analysis, a larger cohort, and the inclusion of a comparator are needed to confirm the early results and should be available shortly. Additionally, a prospective randomized trial setting will be required before propagating this surgical approach as a new treatment option.

## Data Availability

The datasets used and/or analyzed during the current study are available from the corresponding author on reasonable request.

## Abbreviations

ASA: American Society of Anesthesiologists
BMI: Body Mass Index
BM: Biological mesh
FDA: Food and Drug Administration
GmbH: Gesellschaft mit beschraenkter Haftung (Society with restricted liability)
L-RRP: Laparoscopic resection rectopexy
L-SCP: Laparoscopic sacrocolpopexy (synonym for sacrocervicopexy and sacrohysteropexy)
ODS: Obstructed defecation disorder
POP: Pelvic organ prolapse
POP-Q: Pelvic organ prolapse quantification score
SM: Synthetic mesh
